# Effect of *FADS1* rs174556 Genotype on Polyunsaturated Fatty Acid Status: A Systematic Review and Meta-Analysis

**DOI:** 10.1016/j.advnut.2023.01.007

**Published:** 2023-02-05

**Authors:** Wen-Chieh Wu, Pei-Yu Wu, Chien-Yi Chan, Ming-Fen Lee, Chun-Yin Huang

**Affiliations:** 1 Department of Nutrition, China Medical University, Taichung, Taiwan; 2 Department of Nutrition and Health Sciences, Chang Jung Christian University, Tainan, Taiwan

**Keywords:** FADS1, PUFA, meta-analysis, rs174556, single nucleotide polymorphism, systematic review

## Abstract

PUFA status is highly implicated in cognitive development and metabolic disorder-related diseases. Genetic variants of *FADS* genes encoding enzymes that catalyze the rate-limiting steps of PUFA biosynthesis appear to be associated with n-3 and n-6 PUFA contents. Therefore, we conducted the first systematic review and meta-analysis to explore the association of the A-allele carriers of the *FADS1* rs174556 with PUFA status. The PRISMA guidelines were followed. The literature search was conducted up to November 2022 in PubMed, Web of Science, Embase, Cochrane Library, Airiti Library, and CINAHL. The Joanna Briggs Institute checklists were used to assess the methodological quality. The correlation with 95% CIs was determined by a random-effect meta-analysis. Eleven studies that met the inclusion criteria and acceptable quality were included in this systematic review. The data on PUFA contents were collected when they were mainly analyzed using blood samples and breast milk. Results of the meta-analysis on eight studies (one randomized controlled trial, one cohort study, and six cross-sectional studies) showed that the A-allele carriers of rs174556 were significantly negatively correlated with the concentrations of AA (*P* = 0.001), EPA (*P* = 0.004), and DHA (*P* = 0.025). However, ALA and LA were not associated with the A-allele carriers. To clarify the discrepancy, we further divided the studies into blood samples and breast milk subgroups. The subgroup analysis revealed that the A-allele carriers of rs174556 were significantly positively correlated with LA (*P* = 0.031) and negatively correlated with AA (*P* = 0.001), EPA (*P* = 0.036), and DHA (*P* < 0.001) in the blood sample group, but not in the breast milk group. The current meta-analysis proved that the A-allele carriers of the *FADS1* rs174556 appeared to be highly associated with lower concentrations of AA, EPA, and DHA but higher LA in the blood samples. The study has been registered on the International Prospective Register of Systematic Reviews (PROSPERO:CRD42022363978). *Adv Nutr* 2023;x:xx–xx.

**Statement of significance**

This study is the first systematic review and meta-analysis of the association between the *FADS1* rs174556 polymorphisms and PUFA status. The results indicate that A-allele carriers of the *FADS1* rs174556 are negatively associated with AA, EPA, and DHA but positively associated with linoleic acid in the blood samples.

## Introduction

FAPUFAs are a group of important nutrients that are highly implicated in cognitive development and pathogenesis, including cardiovascular disease and metabolic syndrome [[1], [2], [3]]. PUFAs can be divided into two main FA families, n-3 (ω-3) and n-6 (ω-6). The sources of PUFAs include dietary intake and endogenous biosynthesis. Normally, human bodies can synthesize various n-6 and n-3 long-chain PUFAs (LCPUFAs) from two essential FAs, ALA and LA, using FA desaturase (FADS) 1, also known as delta-5 desaturase (D5D), FADS2, also known as delta-6 desaturase (D6D), and elongase [4].

FADS1 and FADS2 catalyze the rate-limiting steps of the PUFA biosynthetic pathway that generates AA, EPA, and DHA [5]. The metabolism of n-6 and n-3 FA families usually compete for the use of limited desaturases and elongases, in which both FADS1 and FADS2 have a higher preference for the n-3 FA metabolism. Intake of vegetable oils rich in n-6 FAs significantly elevates the n-6/n-3 ratio, which in turn affects the conversion rate of ALA to n-3 LCPUFA. Accordingly, maintaining an optimal n-6/n-3 FA ratio at 1:1–1:2 is recommended [6].

FADS1 and FADS2 are encoded by the *FADS1 FADS2* gene cluster located on chromosome 11 (11q12.2-13.1) and arranged head-to-head [7,8]. FADS genetic variants appear to be associated with n-3 and n-6 PUFA proportions in human plasma, tissues, and milk. A study analyzed *FADS1* rs174561, *FADS2* rs174575, and intergenic rs3834458 single nucleotide polymorphisms (SNP) in 309 women from the KOALA Birth Cohort Study in the Netherlands indicated that DHA proportions in plasma phospholipids were lower in women homozygous for the minor allele than those for the major allele [9]. In addition, the FA composition of breast milk was influenced by the genotypes of the rs174553, rs99780, rs174575, and rs174583 in the *FADS1 FADS2* gene cluster, with significantly lower 14:0, AA, and EPA but higher 20:2(n-6) in the minor allele homozygotes of rs174553 (GG), rs99780 (TT), and rs174583 (TT) and lower AA, EPA, 22:5 (n-3), and DHA in the minor allele homozygotes of rs174575 (GG) [10]. Likewise, an SNP (rs1535) and 2-locus haplotypes (rs3834458-rs1535, rs1535-rs174575) in the *FADS2* gene and a 2-locus constructed haplotype (rs174547- rs174553) in the *FADS1* gene were associated with the concentrations of γ-LA and AA in the breast milk of Chinese Han lactating women [11]. In a previous study, we also demonstrated that Taiwanese mothers with high genetic risk (<2 minor alleles in *FADS2* rs1535 and FADS2/3 rs174448) as well as low DHA intake (<200 mg DHA/d) had reduced milk DHA proportions [12]. Together, these data support the effect of *FADS* SNP genotypes on the content of PUFA in humans.

Increasing evidence suggests the physiological significance of the rs174556 SNP of the *FADS1* gene [13,14]. The rs174556 SNP of the *FADS1* gene was reported to affect the maternal and fetal risk factors for cardiovascular diseases [15,16] and FA requirements during pregnancy [14]. The AA genotype of *FADS1* rs174556 presented lower serum concentrations of total cholesterol and LDL than those of the GG genotype, whereas the AA and AG genotypes showed serum higher triglyceride than that of the GG genotype in children [15,16]. Further, the TT genotype of *FADS1* rs174556 presented a higher AA concentration and AA/Dihomo-gamma-linolenic acid index in both plasma and erythrocytes of Alzheimer’s disease patients [13]. The influence of the rs174556 SNP on the FA composition of breast milk has also been reported [17]. Therefore, the current study aimed to integrate existing studies of the FADS1 rs174556 into a meta-analysis tool to define the role of the *FADS1* rs174556 SNP, especially the A-allele carriers, in the PUFA contents, mainly in the blood samples and breast milk.

## Methods

This systematic review and meta-analysis followed the PRISMA guidelines [18]. The protocol for this review was registered at PROSPERO (registration no. CRD42022363978) with no amendment.

### Selection criteria

The inclusion criteria for the study were as follows: *1*) Participants: healthy children and adults of any gender and age around the world. *2*) Study styles were either cross-sectional study, randomized controlled trial (RCT), cohort study, or case-control study. *3*) Information needed to be clearly described, including the number of different *FADS* rs174556 genotypes, concentration or FA percentage of PUFA obtained by standard formula, and tissue of FAs. *3*) Studies written in English or Chinese and published in peer-reviewed journals. Articles were excluded if they were conference abstracts, commentaries, editorials, and letters to the editor, protocols, and review articles.

### Search strategy

The databases, PubMed (National Center for Biotechnology Information, NCBI), Web of Science (Thomson Reuters), Embase (Ovid), Cochrane Library, Airiti Library, and Cumulative Index to Nursing and Allied Health Literature (CINAHL, EBSCO), were searched up to November 2022 to identify studies evaluating the relationship between *FADS* rs174556 SNP and FA status. Keywords and MeSH terms used to search: Fatty Acid Desaturases, Single Nucleotide Polymorphism, PUFA, rs174556. Reference lists were checked for supplement searching.

### Study selection

**FIGURE 1:**
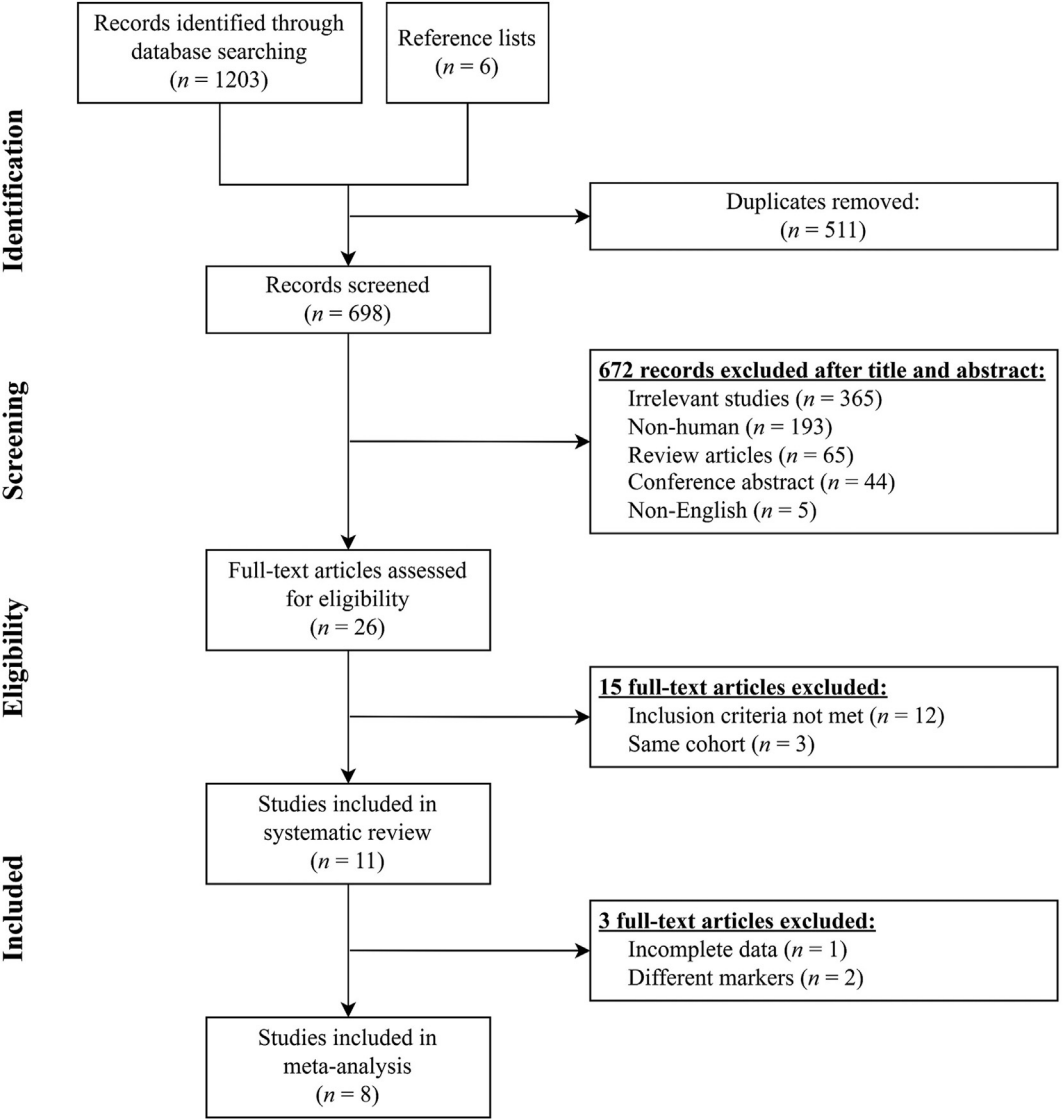
Flow chart of the selection process of the included studies.

Figure 1 shows the flow chart of the study selection. Two authors (W-CW and P-YW) initially identified 1209 publications and imported them into EndNote (Clarivate Analytics) to group the results. However, 511 publications were eliminated due to duplicates.

A total of 672 publications were removed following the title and abstract screening. Then, 26 articles were retained for the full-text assessment. If multiple publications were invented from the same study cohort, only the publication with the most detailed information for outcomes was included. Eleven studies met the inclusion criteria and were included in this systematic review. Among these publications, two were RCTs, one was a cohort study, one was a case-control study, and seven were cross-sectional studies. The data on PUFA contents were mainly collected when they were analyzed using blood samples (including plasma and red blood cells) and breast milk. Two studies that used different tissues, such as adipose tissue [19] and cheek cell glycerophospholipids [20], were excluded from this meta-analysis. However, one of these studies had incomplete data [17]. Finally, eight studies (one RCT, one cohort study, and six cross-sectional studies) were included in this meta-analysis.

### Data extraction

Data from the included studies were extracted to assess study quality and evidence synthesis using a standardized data extraction tool suggested by the Cochrane Collaboration (https://dplp.cochrane.org/data-extraction-forms). The author (W-CW) extracted the data from each selected article. Extracted data covered the study author(s), year and country of publication, participant’s average age, sample size, study methodology, SNP genotypic and allele frequency, PUFA status, and the main conclusions presented. The accuracy of the extracted data was verified by all the authors (W-CW, P-YW, C-YC, M-FL, and C-YH). The missing data were handled by contacting study investigators for unreported data or additional details. Studies with incomplete or missing information were not included in the meta-analysis, but they were discussed in the systematic review. The means of recording data were in a Microsoft Excel 2019 spreadsheet.

### Quality assessment

The studies were retained for the full-text assessment using a 3-point scale (0 = not relevant, 1 = unsure, and 2 = relevant). Two reviewers (W-CW and P-YW) independently assessed the relevance, resolved any disagreement, and consulted a third reviewer (C-YH) when a consensus could not be reached.

The Joanna Briggs Institute (JBI) checklist [21] was employed to evaluate the methodological quality of the studies included by two reviewers (W-CW and P-YW). Studies with more than half “yes” answers in the JBI checklists were considered good quality and included in this systematic review. The third reviewer (C-YH) resolved any discrepancies that occurred. JBI checklists contain 8, 13, 11, and 10 questions for cross-sectional, RCTs, cohort, and case-control studies, respectively. Overall, all cross-sectional, RCTs, cohort, and case-control studies had “yes” answers for more than half of the questions on the JBI checklists. Therefore, all 11 studies exhibited appropriate quality and acceptable reporting bias (Table 1).

**TABLE 1:** Descriptive characteristics of the included studies

Study (reference)	Study design	Year and country	Study cohort	Study participants	Sample size, *n*	aged	Genotyping method	PUFA detection methods (tissue)	PUFA status	Quality score
Koletzko et al. [24]	Cross-sectional study	2011, United Kingdom	The ALSPAC birth cohort study	Pregnant woman	6524	28.57 ± 4.59	MassARRAY system	GLC (red blood cell phospholipids)	Minor allele carriers (A) of rs174556 were positively associated with precursor FAs and negatively associated with LCPUFAs and product: substrate ratios of n-6 and n-3 pathways.	8/8
Gonzalez-Casanova et al. [23]	Cross-sectional study	2016, Mexico	The POSGRAD birth cohort study	Pregnant woman	140	26.4 ± 4.73	MassARRAY system	Gas chromatograph (plasma)	Minor allele carriers (G) of rs174556 were positively associated with AA and DHA plasma concentrations.	7/8
Lattka et al. [25]	Cross-sectional study	2011, Germany	The Ulm birth cohort study	Postpartum women	711	31.29 ± 4.76	MassARRAY system	GLC (breast milk)	Minor allele carriers (A) of rs174556 were significantly associated with lower AA concentrations and AA/DGLA ratios.	6/8
Rzehak et al. [27]	Cohort study	2010, Netherlands and Germany	The KOALA and the LISA birth cohort study	Postpartum women and children	862	2	MALDI-TOF MS	Gas chromatographic (plasma phospholipid and glycerophospholipids)	Minor allele carriers (A) of rs174556 were significantly associated with higher LA and DGLA concentrations and lower GLA, AA, ADA, DPA, and DHA concentrations.	6/11
Muc et al. [22]	Cross-sectional study	2015, Denmark	The COPSAC 2000 birth cohort study	Postpartum women and children	111	29.73 ± 4.25	High-throughput genome-wide SNP genotyping (Illumina)	GLC (breast milk)	Minor allele carriers (A) of rs174556 were significantly associated with lower AA concentrations.	6/8
Meldrum et al. [33]	Double-blind RCT	2018, Australia	The IFOS study	Children	123	3–6 M	MALDI-TOF MS	GLC (plasma)	Minor allele homozygotes carriers (AA) of rs174556 in fish oil intervention were significantly associated with higher DHA concentrations.	9/13
Muzsik et al. [32]	Cross-sectional study	2018, Poland	A clinical trial	Women in the postmenopausal period and under 70 y old	125	60.7 ± 5.1	real-time PCR	GC (red blood cell)	There were no associations between FAs in red blood cell concentrations and the rs174556 polymorphisms.	8/8
Mychaleckyj et al. [17]	Cross-sectional study	2018, Bangladesh	The PROVIDE study and the CRYPTO study	Postpartum women	1142	-	GWAS genotyping (Illumina)	GC (breast milk)	Major allele carriers (A) of rs174556 were significantly associated with higher AA concentrations.	7/8
Martínez-Zaldívar et al. [20]	RCT	2019, Germany and Spain	The NUHEAL trial	Pregnant women and children	240	8–9.5	MALDI-TOF MS	GC(cheek cell glycerophospholipids)	Minor allele carriers (A) of rs174556 were associated with higher LA and ALA concentrations in children.	11/13
Aslibekyan et al. [19]	Case-control study	2012, Costa Rica	The Costa Rica study	First myocardial infarction under 75 y old	1032	58.1 ± 11.0	SNPlex genotyping system	GLC (adipose tissue)	Minor allele carriers (A) of rs174556 were significantly associated with higher ALA, EDA, DGLA, and ETA concentrations and lower GLA, AA, and EPA concentrations in adipose.	8/10
Rzehak et al. [31]	Cross-sectional study	2009, Germany	The BVS-II study	the German-speaking population+13 to 80 y old	535	13–80	MassARRAY system	GC (erythrocyte membranes)	Minor allele carriers (A) of rs174556 were significantly associated with higher DGLA concentrations and lower AA and ADA concentrations.	5/8

AA, arachidonic acid; ADA, adrenic acid; ALA, alpha-linolenic acid; ALSPAC, Avon Longitudinal Study of Parents and Children; BVS-II, Bavarian Nutrition Survey II; COPSAC, Copenhagen Prospective Studies on Asthma in Childhood; CRYPTO, Cryptosporidiosis in Bangladesh; DGLA, dihomo-γ-linolenic acid; DHA, docosahexaenoic acid; DPA, docosapentaenoic acid; EPA, eicosapentaenoic acid; ETA, eicosatetraenoic acid; GLA, γ-linolenic acid; GWAS, Genome-Wide Association Studies; IFOS, Infant Fish Oil Supplementation Study; KOALA, Kind, Ouders en gezondheid: Aandacht voor Leefstijl en Aanleg; LA, linoleic acid; LCPUFAs, long-chain PUFAs; LISA, Influences of Lifestyle related Factors on the Immune System and the Development of Allergies in Childhood; MALDI-TOF MS, matrix-assisted laser desorption ionization-time of flight MS; NUHEAL, Nutraceuticals for a Healthier Life; PCR, polymerase chain reaction; POSGRAD, Prenatal ω-3 Supplementation on Child Growth and Development; PROVIDE, Performance of Rotavirus and Oral Polio Vaccines in Developing Countries; RCT, randomized controlled trial; SNP, single nucleotide polymorphism.

### Statistical methods

The meta-analysis was performed by the Comprehensive Meta-Analysis version 2.0. The correlation and 95% CIs were used to identify the association of *FADS* rs174556 SNP with FA status. In one study [22], the mean and SD were calculated from medians and the IQR as the following equation: mean = median; SD=IQR/1.35. In addition, in another study [20] we calculated the SDs from 95% CIs using the following equation: SE=(upperlimit−lowerlimit)/3.92. For one study [23], SEs were calculated from SDs as the following equation:$SE=SD/\sqrt{n}$. Because some studies [17,[24], [25], [26], [27]] used log-transformed data like means, Beta coefficient, SDs, and SEs, we converted those data into new values using the following equation [28,29]:

$$mean=\exp\left(\mu +\frac{\sigma^{2}}{2}\right)$$

$$SD=\sqrt{(\exp\left(\sigma^{2}\right)-1)\times \exp\left(2\mu +\sigma^{2}\right)}$$

(μ=logmean,σ=logSD)

Of studies included in the meta-analysis, heterogeneity between the studies in effect measures was assessed using the I^2^ test. Because of the high heterogeneity of studies, this meta-analysis used the random effects model [30]. The risk of bias and publication bias was evaluated as evidence of certainty. We also used funnel plots and the Begg and Mazumdar rank test to evaluate the publication bias with the significance level set at *P* < 0.05. In addition, sensitivity analysis (Supplemental Figure 1) was achieved by a sequential, systematic meta-analysis by excluding 1 study at a time.

## Results

### Study characteristics

The characteristics of studies included in the analysis are summarized in Table 1. These 11 studies were published between 2009 and 2019. Seven of those were cross-sectional studies [17,[22], [23], [24], [25],^31^,^32^], 2 were RCTs [20,33], 1 was a case-control study [19], and one was a cohort study [27]. However, one of these studies had incomplete data [17], and two used different markers (tissue) [19,20]; therefore, these three studies were excluded, and the remaining seven studies were undergone meta-analysis. A total of 11,545 participants across different ethnic populations were from the following 10 countries: Germany [20,25,27,31], the United Kingdom [24], Mexico [23], the Netherlands [27], Denmark [22], Australia [33], Poland [32], Bangladesh [17], Spain [20], and Costa Rica [19]. The majority of the participants were women (pregnant or postpartum women) and their children, except for one case-control study [19] and one cross-sectional study [31] with both genders of participants. The majority of the participants were in the age range of 20–40 y old; however, the mean age of the participants in two studies was ≥59 y old [19,32]; 3 studies recruited children under 10 y old [20,27,33], and one study had participants in a vast range of age from 13–80 y old [31]. Last, all studies determined the number of PUFAs using gas (-liquid) chromatography.

### Effect of *FADS1* rs174556 SNP on the ALA concentration

**FIGURE 2:**
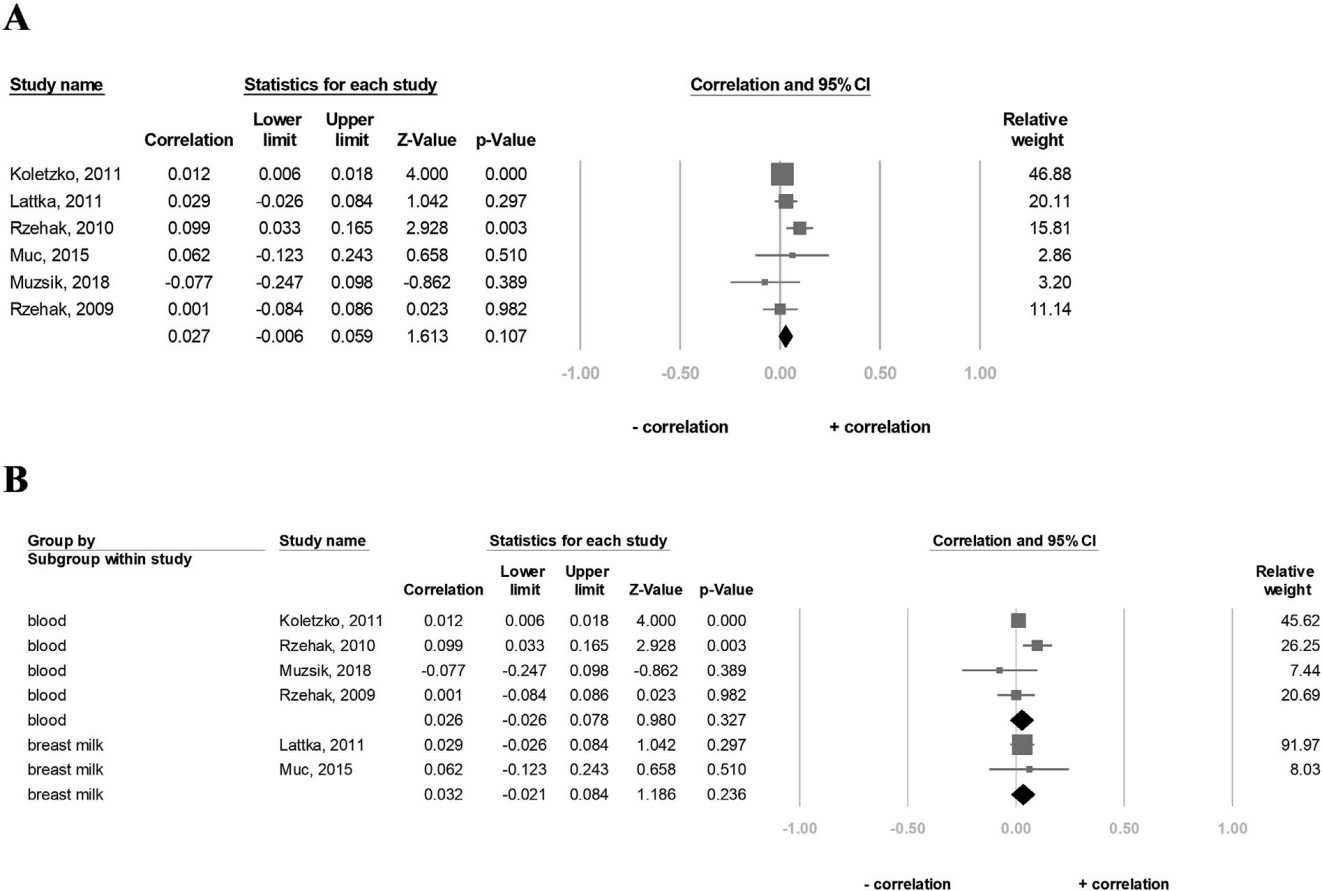
Forest plot of the correlation between the A-allele carriers of the FADS1 rs174556 and ALA status for (A) six studies and (B) subgroup analysis of blood tissue and breast milk. 95% CI and study weights were provided for each study. ALA, α-linolenic acid; FADS1, FA desaturase 1.

A total of 8324 participants in the six studies were included to evaluate the association between the A-allele carriers of the *FADS1*-rs174556 and ALA concentrations. The result showed that the ALA concentration in the A-allele carriers was not significantly different from that in the G-allele carriers (correlation: 0.027, 95% CI: −0.006 to 0.059, *P* = 0.107, I^2^ = 39.6%) (Figure 2A). Further, subgroup analysis was performed for breast milk and blood (blood, including red blood cells and plasma), and the results indicated that there was no significant correlation between the A-allele carriers and ALA concentrations in either the blood sample group (correlation: 0.026, 95% CI: −0.026 to 0.078, *P* = 0.327, I^2^ = 60.8%) or the breast milk group (correlation: 0.032, 95% CI: −0.021 to 0.084, *P* = 0.236, I^2^ = 0%) (Figure 2B). Besides, the sensitivity analysis also revealed that the ALA concentration was not significantly associated with the A-allele carriers after excluding 1 study at a time (Supplemental Figure 1A).

### Effect of *FADS1* rs174556 SNP on the LA concentration

**FIGURE 3:**
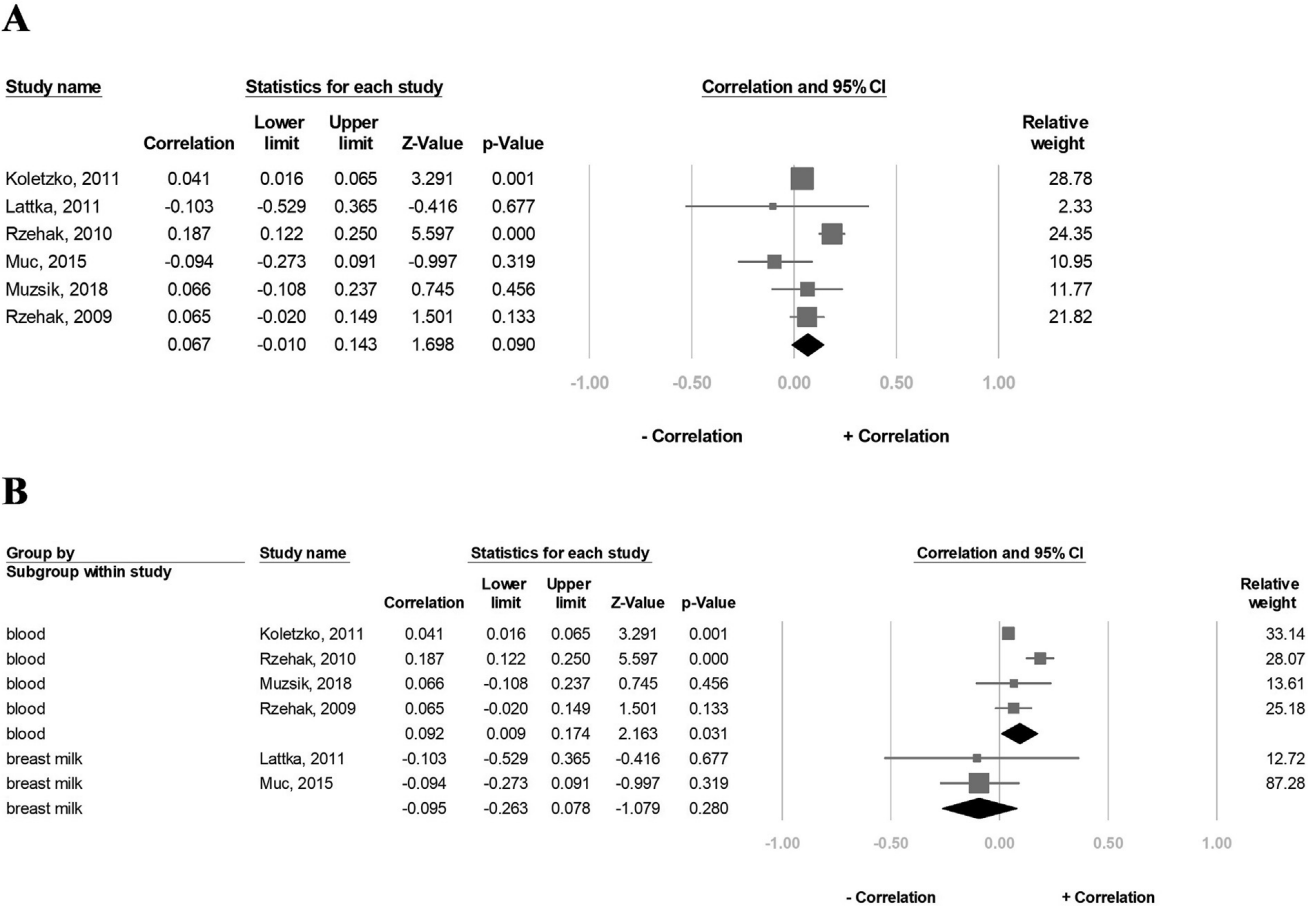
Forest plot of the correlation between the A-allele carriers of the FADS1 rs174556 and LA status for (A) six studies and (B) subgroup analysis of blood tissue and breast milk. 95% CI and study weights were provided for each study. FADS1, FA desaturase 1; LA, linoleic acid.

A total of 8324 participants in six studies were included in this analysis. The result showed that the LA concentration and the A-allele carriers were not significantly correlated (correlation: 0.067, 95% CI: −0.010 to 0.143, *P* = 0.090, I^2^ = 39.6%) (Figure 3A). The sensitivity analysis also showed that the LA concentration was not significantly associated with the A-allele carriers (Supplemental Figure 1B). Nonetheless, in the subgroup analysis, the LA concentration significantly positively correlated with the A-allele carriers in the blood sample group (correlation: 0.092, 95% CI: 0.009–0.174, *P* = 0.031, I^2^ = 82.4%) but not in the breast milk group (correlation: −0.095, 95% CI: −0.263 to 0.078, *P* = 0.280, I^2^ = 0%) (Figure 3B).

### Effect of *FADS1* rs174556 SNP on the AA concentration

**FIGURE 4:**
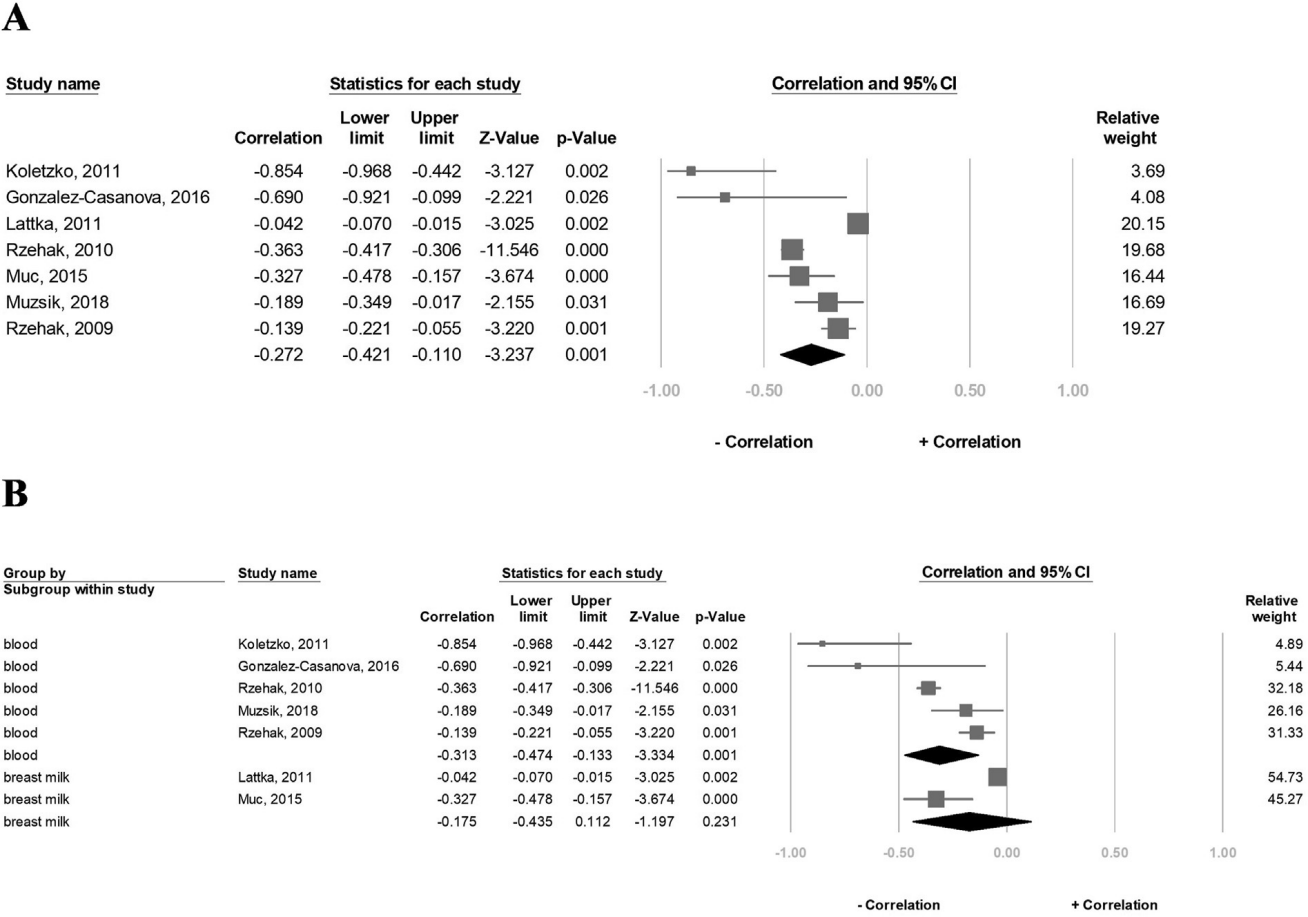
Forest plot of the correlation between the A-allele carriers of the FADS1 rs174556 and AA status for (A) seven studies and (B) subgroup analysis of blood tissue and breast milk. 95% CI and study weights were provided for each study. AA, arachidonic acid; FADS1, FA desaturase 1.

A total of 8464 participants in seven studies were included in the correlation analysis. The result showed that the overall AA concentration was negatively correlated with the A-allele carriers (correlation: −0.272, 95% CI: −0.421 to 0.110, *P* = 0.001, I^2^ = 94.5%) (Figure 4A). Further, results from the subgroup analysis showed a significant negative correlation between the AA concentration and the A-allele carriers in the blood sample group (correlation: −0.313, 95% CI: −0.474 to −0.133, *P* = 0.001, I^2^ = 86.1%), but not in the breast milk group (correlation: −0.175, 95% CI: −0.435 to 0.112, *P* = 0.231, I^2^ = 90.1%) (Figure 4B). After excluding 1 study at a time, the sensitivity analysis also indicated that a significantly negative association remained between the overall AA concentration and the A-allele carriers (Supplemental Figure 1C).

### Effect of *FADS1* rs174556 SNP on the EPA concentration

**FIGURE 5:**
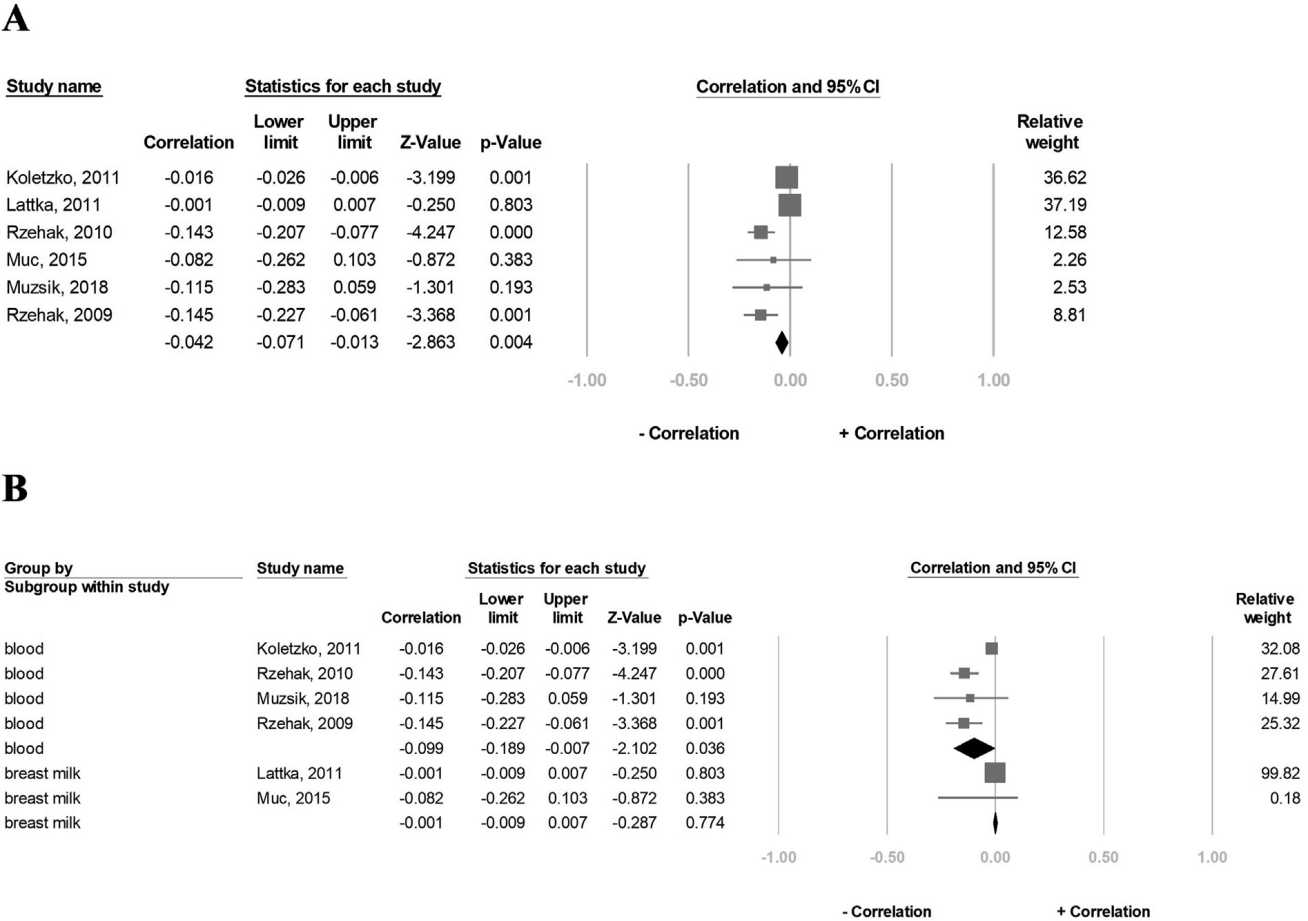
Forest plot of the correlation between the A-allele carriers of the FADS1 rs174556 and EPA status for (A) six studies and (B) subgroup analysis of blood tissue and breast milk. 95% CI and study weights were provided for each study. EPA, eicosapentaenoic acid; FADS1, FA desaturase 1.

A total of 8324 participants in six studies were included in the correlation analysis. The result showed that the overall EPA concentration negatively correlated with the A-allele carriers (correlation: −0.042, 95% CI: −0.071 to 0.013, *P* = 0.004, I^2^ = 85.2) (Figure 5A). Further, the subgroup analysis revealed a significant negative correlation between the EPA concentration and the A-allele carriers in the blood sample group (correlation: −0.099, 95% CI: −0.189 to −0.007, *P* = 0.036, I^2^ = 87.3), but not in the breast milk group (correlation: −0.001, 95% CI: −0.009 to 0.007, *P* = 0.774, I^2^ = 0%) (Figure 5B). In the sensitivity assessment, the overall EPA concentration remained negatively associated with the A-allele carriers (Supplemental Figure 1D).

### Effect of *FADS1* rs174556 SNP on the DHA concentration

**FIGURE 6:**
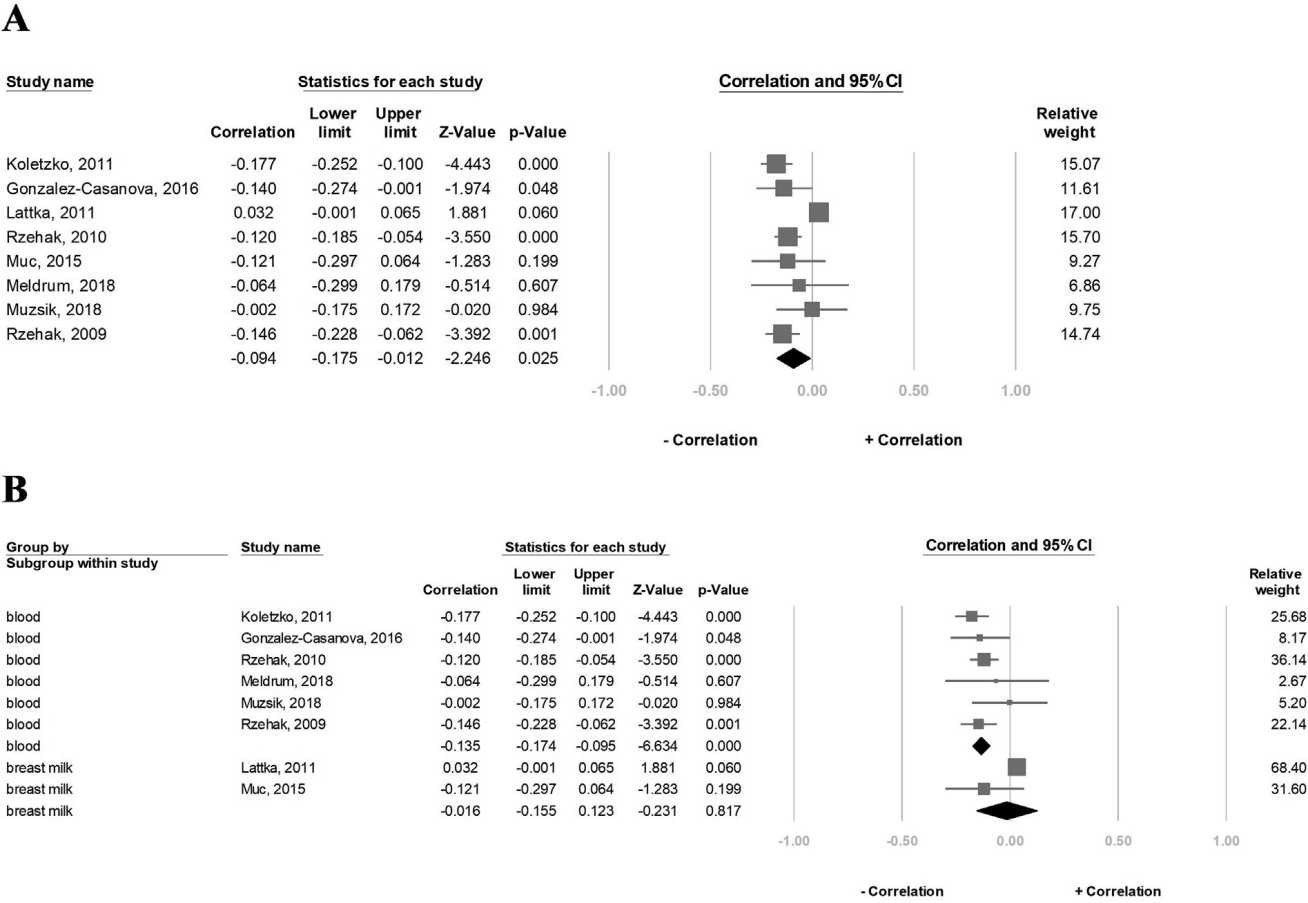
Forest plot of the correlation between the A-allele carriers of the FADS1 rs174556 and DHA status for (A) 8 studies and (B) subgroup analysis of blood tissue and breast milk. 95% CI and study weights were provided for each study. DHA, docosahexaenoic acid; FADS1, FA desaturase 1.

Last, 8489 participants in eight studies were evaluated for the association between carriers of the A-allele of the FADS1-rs174556 and the DHA concentrations. The result revealed that the DHA concentration was significantly negatively correlated with the A-allele carriers (correlation: −0.094, 95% CI: −0.175 to 0.012, *P* = 0.025, I^2^ = 84.3%) (Figure 6A). However, in the sensitivity assessment, the negative association between the DHA concentration and the A-allele carriers became insignificant after excluding 1 study at a time (Supplemental Figure 1E). The subgroup analysis further revealed that the DHA concentration in the blood sample group was significantly negatively correlated with the A-allele carriers (correlation: −0.135, 95% CI: −0.174 to −0.095, *P* < 0.001, I^2^ = 0%), but not in the breast milk group (correlation: −0.016, 95% CI: −0.155 to 0.123, *P* = 0.817, I^2^ = 60.7%) (Fig. 6B).

### Publication bias

Supplemental Figure 2 shows the funnel plot of the correlation between the A-allele carriers of the *FADS1* rs174556 and 5 FAs status. There appeared an asymmetry in some funnel plots (AA, EPA, and DHA) but not in the other two funnel plots (ALA and LA). Nevertheless, publication bias was absent in the analysis according to the Begg and Mazumdar rank test [*P* = 1.00 (ALA and EPA), 0.71 (LA), 0.23 (AA), 0.53 (DHA)].

## Discussion

The current study was the first meta-analysis research to investigate the effect of *FADS1* rs174556 SNP on PUFA composition in human tissues. Our results revealed that the population with the rs174556 A-allele exhibited significantly lower n-6 and n-3 LCPUFA products, AA, EPA, and DHA, and higher 18-carbon FA substrate, LA in the blood, which is highly implicated in impairing cognitive function or increased the risk of cardiovascular and metabolic syndrome [[1], [2], [3]].

The significance of the *FADS1* rs174556 SNP with pathological manifestations has been addressed where rs174556 SNP was reported to be associated with asthma [34,35], pregnancy duration and birthweight [14], and blood concentrations of lipids[15,16]. Daily margarine intake (high content of n-6 LA) was significantly associated with asthma in the GG genotype. The authors suggested that for G-allele carriers of the *FADS1*-rs174556, a higher percentage of LA is metabolized to AA which can act as a substrate of inflammatory eicosanoids [34]. The AA genotype of the *FADS1*-rs174 556’s mothers had 2-d shorter pregnancies (*P* = 0.035), and their children were 140 g lighter (*P* = 0.006) than those of the GG genotype’s mothers [14].

Functional intron polymorphisms may occur and affect the transcriptional activity when mutations in crucial elements within introns are implicated in RNA splicing [36]. The rs174556 SNP resides in the intron 2 of the *FADS1* gene, <100 base pairs 3’ to exon 2, and within a region of H3K4Me1 and H3K27Ac marks indicating that this locus may play a critical role in alternative splicing, splicing frequency, and epigenetic modulation of transcription activity. Besides, rs174556 is a locus for several gene transcripts in multiple tissues, including both *FADS1* and *FADS2* [17]. As shown in Table 1, the *FADS1* rs174556 genotype significantly affected desaturase activity in some studies [24,25]. When the actions of D5D and D6D enzymes were estimated using the ratio of FA products to precursors in tissues [37], Women with the A-allele of the *FADS1* rs174556 had lower FADS desaturase activity. However, one cannot exclude the possibility that the decrease in enzyme activity may be directly due to the reduced amount of enzyme.

Results of the meta-analysis indicated that the impact of genetic variation on AA (correlation: −0.272, *P* = 0.001) appeared much higher than that of DHA (correlation: −0.094, *P* = 0.025) and EPA (correlation: −0.042, *P* = 0.004). This phenomenon may be explained by the fact that the human tissues contained relatively higher AA content to EPA and DHA (AA, 6.09% and 0.44% of total FAs in red blood cells and breast milk, respectively) [24,25]. When the original AA content in the blood and breast milk is high, the AA reduction change may also be significant, reflecting the consistency observed in Figure 4A. In contrast, DHA (2.01% and 0.22% of total FAs in red blood cells and breast milk, respectively) and EPA (0.24% and 0.06% of total FAs in red blood cells and breast milk, respectively) originally existed in the blood and breast milk in a relatively low amount [24,25]; therefore, an accurate detection may become difficult. Besides, because of the wide range of the DHA and EPA values among individuals, researchers often carried out logarithmic (log) transformations on DHA and EPA values. Therefore, reducing the amount of DHA and EPA in the A-allele carriers of the *FADS1*-rs174556 becomes relatively limited [24,25,27].

Also, we carried out the subgroup analysis to evaluate the tissues that were affected by the *FADS1* rs174556 SNP. The results showed that the FAs in the blood sample group (plasma or red blood cells) were more significantly affected by the rs174556 variation. The A-allele of rs174556 showed a significant positive correlation with ALA and LA concentrations and a significant negative correlation with AA, EPA, and DHA in blood. However, no significant association was found between the PUFA content of the breast milk and A-allele carriers of the *FADS1*-rs174556. Because only two publications in breast milk-related studies were incorporated into the analysis, the results may be less representative. However, it is worth noting that both diet and genotype and their interaction contribute to the FA composition of breast milk [9,12]. It has been reported that DHA intake and *FADS* genotype (rs174561, 174575, rs3834458, and rs1535) significantly affected the proportion of DHA in erythrocyte, plasma phospholipids and breast milk of postpartum women [9]. The amount of plasma DHA was positively correlated with the intake of fish and fish oil, but the amount of DHA in breast milk did not increase with the increased intake of fish and fish oil in the minor allele group of the *FADS* SNP. Nonetheless, more studies on the effects of the *FADS1* rs174556 SNP and its combination with diet on the FA composition of breast milk would clarify the picture.

The allele frequency of the *FADS1*-rs174556 has an ethnic discrepancy. Most of the studies included in the current meta-analysis had Europeans as the main subjects, and the major/minor alleles of the *FADS1*-rs174556 were G/A. In contrast, American Mexicans [23] and Asian Bengalis [17], as the main subjects in two studies, had A/G as the major/minor alleles of the *FADS1*-rs174556, respectively. This is in agreement with the information in the SNP database of the NCBI [38] that the A-allele frequency of rs174556 in Europeans was 29.6%, whereas the A-allele frequency in Asians and Mexicans was 66.5% and 60.7%, respectively. It is worth noting that regardless of the A-allele of rs174556 as the major allele (as in Mexicans) or the minor allele (as in most Europeans), A-allele carriers of rs174556 appeared to be less favorable for AA, EPA, and DHA in the tissues tested. This observation was similar to that of other SNP of the *FADS* gene. Although the major allele of rs1535 SNP differs between Caucasians (A) and Southern Han Chinese (G) [12], carriers of the G-allele had lower FADS desaturase activity than that of the A-allele.

This systematic review and meta-analysis had some limitations. First, there may be a publication bias in this analysis because only studies with favorable results were easily searched, whereas literature with poor results or interruptions in follow-up might not have been identified. Furthermore, the number of articles recruited in this meta-analysis was relatively small, which may limit the meta-analysis, sensitivity analysis, and subgroup analysis results. Third, the literature included in the analysis was relatively old such that most of the literature was from >5 y ago. Fourth, we did not investigate the interaction between genes and diet because the studies included in this meta-analysis did not have enough raw data, including dietary information. Nonetheless, the current analysis provided indisputable evidence connecting the significance of the *FADS1* rs174556 SNP to PUFA contents that might inspire future investigations in detailed subgroup analysis, such as long-term RCTs or cohort studies on various tissues, in different populations to overcome the limitations.

In conclusion, the current meta-analysis indicated that the A-allele carrier of the *FADS1* rs174556 may exhibit altered PUFA metabolism with lower concentrations of AA, EPA, and DHA but higher LA in the blood, in particular.

## Funding

This research was funded by the Ministry of Science and Technology, Taiwan, grant number MOST 111-2320-B-039-020-MY3, and China Medical University, Taiwan, grant number CMU107-S-22 and CMU109-MF-53.

## Author disclosures

The authors report no conflicts of interest.

## Acknowledgments

The authors’ responsibilities were as follows: W-CW and C-YH conceptualized the study. W-CW, P-YW, C-YC, M-FL, and C-YH performed the literature search, data extraction, and verification. W-CW and P-YW contributed to the statistical analysis and data interpretation. W-CW, P-YW, C-YC, M-FL, and C-YH participated in writing and original draft preparation. W-CW, M-FL, and C-YH assisted in writing, reviewing, and editing. All authors have read and agreed to the published version of the manuscript.

### Data availability

The data that support the findings of this study are available from the corresponding author upon request.

## Appendix A Supplementary data

Multimedia component1 Multimedia component1
