# Supplementary material for: Effect of FADS1 rs174556 Genotype on Polyunsaturated Fatty Acid Status: A Systematic Review and Meta-Analysis
Source: Adv Nutr. 2023 Feb 5;14(2):352–62. doi: 10.1016/j.advnut.2023.01.007 (PMC10229383; doi:10.1016/j.advnut.2023.01.007)
Supplement: Multimedia component1 [file mmc1.docx]

**Effect of FADS1 rs174556 genotype on polyunsaturated fatty acid (PUFA) status: A systematic review and meta-analysis**

Wen-Chieh Wu^#^& Pei-Yu Wu^#^

# These two authors contributed equally to the work

1. ALA

1. LA

1. AA

1. EPA

1. DHA

**Supplementary Figure 1** **Sensitivity analysis of the meta-analysis.** (A-B, D) The sensitivity analysis for 6 studies on the correlation between the A-allele carriers of the FADS1 rs174556 and status of (A) ALA status, (B) LA status, and (D) EPA. (C) The sensitivity analysis of the correlation between the A-allele carriers of the FADS1 rs174556 and AA status for (C) 7 and (E) 8 studies

Abbreviation: ALA, alpha-linolenic acid; LA, linoleic acid; AA, arachidonic acid; EPA, eicosapentaenoic acid; DHA, docosahexaenoic acid; CI, confidence intervals

A ALA B LA

|   C AA |   D EPA |
| --- | --- |
|   E DHA |  |
|  |  |

**Supplementary Figure 2 Funnel plots of the publication bias.** (A-B, D) The Funnel plots for 6 studies on the correlation between the A-allele carriers of the FADS1 rs174556 and status of (A) ALA status, (B) LA status, and (D) EPA. (C) The Funnel plots of the correlation between the A-allele carriers of the FADS1 rs174556 and AA status for (C) 7 and (E) 8 studies

Abbreviation: ALA, alpha-linolenic acid; LA, linoleic acid; AA, arachidonic acid; EPA, eicosapentaenoic acid; DHA, docosahexaenoic acid
